# Causal relationship between endometriosis and inflammatory bowel disease: A Mendelian randomization analyses

**DOI:** 10.1002/ctm2.1496

**Published:** 2024-01-18

**Authors:** Yan Dang, Shutian Zhang

**Affiliations:** ^1^ Department of Gastroenterology, Beijing Friendship Hospital, Capital Medical University, National Clinical Research Center for Digestive Disease Beijing China

Dear Editor,

Endometriosis (EMS), a chronic gynecological disorder characterized by the presence of endometrial‐like tissue outside the uterus, is intricately understood. The presence of an inflammatory environment outside the pelvic region has prompted researchers to reconsider EMS as an immunity‐associated systemic disorder.^1^ The term inflammatory bowel disease (IBD) refers to inflammation in the gastrointestinal tract, traditionally categorized into ulcerative colitis (UC) and Crohn's disease (CD). The coexistence of IBD and EMS has been documented.^2^ Craninx et al. reported histological features of EMS in CD patients undergoing surgical resection without a preoperative EMS diagnosis.^3^ Deep infiltrating EMS and posterior adenomyosis were significantly more frequent in patients with IBD. Additionally, Jess et al. reported that women with EMS were 50% more likely to suffer from IBD than women in general.^4^ However, in observational studies, potential biases from residual confounding and reverse causality affect the inference of cause‐effect relationships. The question of whether IBD induces or promotes EMS, or vice versa, remains unclear.

Mendelian randomization (MR) is a methodological approach in epidemiological research that assesses the potential causality of a risk factor or modifiable exposure and clinical outcome by utilizing genetic instruments. Genetic variants, unalterable and randomly assigned during human germ cell formation, enable MR to impartially evaluate exposure effects, avoiding common confounding or reverse causality issues in observational studies (Figure 1A).^5^ The current understanding of the relationship between EMS and IBD is confined to observational studies, incapable of establishing a direct causal relationship. Additionally, conducting randomized controlled trials is not feasible. Therefore, we applied a two‐sample MR approach to elucidate the causality between the two.

FIGURE 1(A) The basic principles of mendelian analysis (MR). Instrumental variables (IVs) have to fulfill three principal assumptions: 1. they are closely related to the risk factor of interest; 2, they are not correlated with any potential confounders influencing the outcomes; 3, they affect the outcomes only via the risk factor; (B) workflow of the MR study design. Single nucleotide polymorphisms (SNPs) were extracted as IVs if reaching the genome‐wide association studies (GWAS) P < 5E‐8 and were further clumped to ensure the independency of instruments (clumping criteria: linkage disequilibrium [LD] *r* ^2^ = 0.001 and window size = 10,000 kb). Also, SNPs with P < 1E‐5 in the PhenoScanner database would be deleted in the following analysis. (C) The results of MR analysis taking endometriosis (EMS) as exposure to estimate their causal effect on inflammatory bowel disease (IBD), ulcerative colitis (UC) and Crohn's disease (CD). (D) The results of MR analysis of EMS (Sapkota Y) on IBD, UC and CD. ASRM, American Society for Reproductive Medicine; MR‐PRESSO, mendelian Randomization Pleiotropy RESidual Sum and Outlier; OR, odds ratio; CI, confidence interval.

**Figure:**
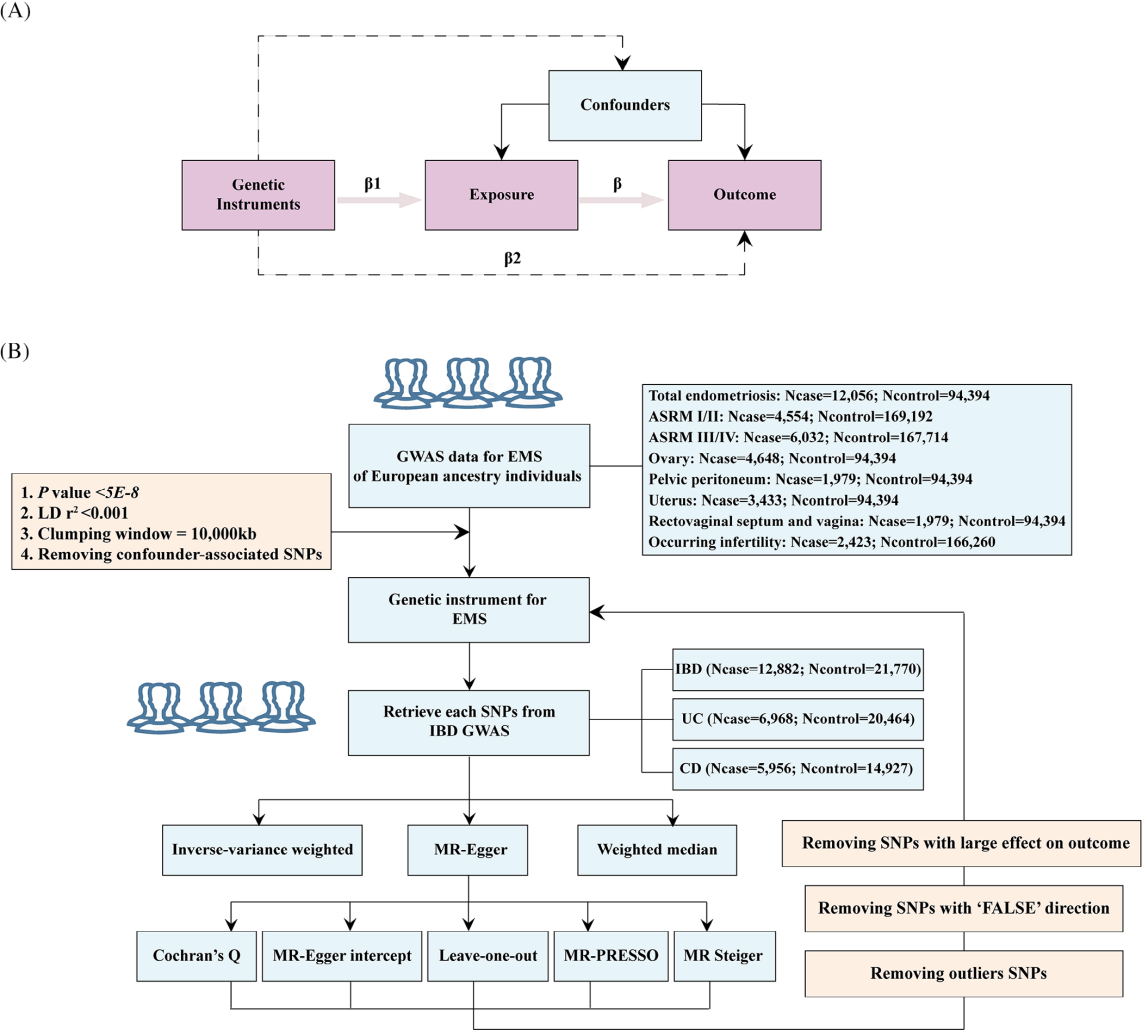

**Figure:**
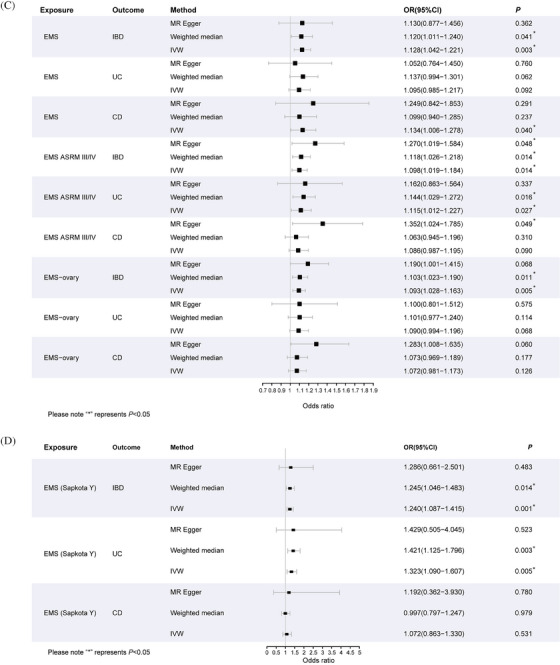

EMS and IBD large‐scale genome‐wide association study (GWAS) summary results are presented in Tables 1A and 1B, with no sample overlap between them.^6^, ^7^ Figure 1B displays the selection process for instrumental variables (IVs) of EMS. Detailed information about the finally selected IVs is listed in Table S1.

**TABLE 1A:** Description of the outcome IBD.

	Sample size			
Outcome (total and subgroups)	case	control	GWAS Catalog accession number	PMID
IBD	12 882	21,770	ieu‐a‐31	26192919
UC	5956	14,927	ieu‐a‐32	26192919
CD	6968	20,464	ieu‐a‐30	26192919

Abbreviations: CD, Crohn's disease; GWAS, genome‐wide association studies; IBD, inflammatory bowel disease; UC, ulcerative colitis.

**TABLE 1B:** Description of the exposure EMS.

	Sample size		
Exposure (total and subgroups)	Case	Control	GWAS catalog accession number
Total EMS	12 056	94 394	finn‐b‐N14_ENDOMETRIOSIS
ASRM I/II	4554	169 192	finn‐b‐N14_ENDOMETRIOSIS_ASRM_STAGE1_2
ASRM III/IV	6032	167 714	finn‐b‐N14_ENDOMETRIOSIS_ASRM_STAGE3_4
Ovary	4648	94 394	finn‐b‐N14_ENDOMETRIOSIS_OVARY
Pelvic peritoneum	1979	94 394	finn‐b‐N14_ENDOMETRIOSIS_PELVICPERITONEUM
Uterus	3433	94 394	finn‐b‐N14_ENDOMETRIOSIS_UTERUS
Rectovaginal septum and vagina	1979	94 394	finn‐b‐N14_ENDOMETRIOSIS_RECTPVAGSEPT_ VAGINA
Occurring infertility	2423	166 260	finn‐b‐N14_ENDOMET_INFERT
Total EMS (Sapkota Y)	14 926	189 715	GCST004549

Abbreviations: ASRM, American Society for Reproductive Medicine; EMS, endometriosis; GWAS, genome‐wide association studies.

We employed five different methods for the MR effect estimates. We used inverse‐variance weighted (IVW) linear regression as our primary method. A statistically significant causal association is defined as an adjusted *p* < 0.017, indicating a considerable relationship between the exposure and outcome phenotype. We used a series of sensitivity analyses to address variants heterogeneity and the pleiotropy effect, the flow of all MR analyses can be available in Figure 1B.^8^, ^9^ Another EMS GWAS dataset provided by Sapkota Y was also used to validate our findings. An R language version of 4.2.0 was used for all analyses. R‐based packages ‘TwoSampleMR’ and ‘MRPRESSO’ were applied to conduct MR analyses.^10^ The data visualization was conducted using the ‘TwoSampleMR’ and ‘forestplot’.

In a cohort of people with different subtypes of EMS, MR analysis was carried out to estimate the risk of IBD following EMS. As shown in Table 1C, the genetically predicted EMS increased IBD risk (OR = 1.128, P_IVW_ = 0.003). Subsequent analyses focusing on the main subtypes of IBD, UC, and CD, suggested a causal association between EMS and CD (P_IVW_ = 0.040). However, the statistical significance was lost after applying the Bonferroni adjustment to account for multiple comparisons. When performing MR analysis with different subtypes of EMS as the exposures and IBD as the outcome, a significantly higher risk of IBD was observed only for ovarian EMS (OR = 1.093, P_IVW_ = 0.005). Furthermore, we performed MR analyses of different EMS stages as exposure (as determined by the American Society for Reproductive Medicine [ASRM]). The causality between EMS ASRM III/IV and IBD as a whole is notably significant (OR = 1.098, P_IVW_ = 0.014). However, null significant causal associations are observed between different EMS stages and UC or CD. Additionally, our reverse MR analysis revealed no causal relationship between IBD (exposure) and EMS (outcome) (see Table S2). Another MR analysis, utilizing the GWAS data provided by Sapkota, provides additional support for these findings (Table 1D). Genetically predicted EMS increased the risk of IBD (OR = 1.240, P_IVW_ = 0.001). Although this analysis further suggested the causality of EMS on UC (OR = 1.323, P_IVW_ = 0.005), this conclusion was not consistent with the original analysis. No obvious horizontal pleiotropy or outliers were detected in all MR analyses (*p* > 0.05). Analyses of sensitivity are shown in the Supplementary Figure.

**TABLE 1C:** Causal estimation for the effect of EMS on IBD, UC as well as CD.

Exposure	Outcome	MR methods	Number of SNPs	OR (95% CI)	SE	MR *p*‐value	MR‐Steiger	*p*‐Value heterogeneity	*p*‐Value pleiotropy
EMS_total	IBD	MR Egger	14	1.130 (.877–1.456)	.129	.362	True	.840	.985
		IVW	14	1.128 (1.042–1.221)	.040	.003		.888	
		Weighted median	14	1.120 (1.011–1.240)	.054	.041			
EMS_total	UC	MR Egger	13	1.052 (.764–1.450)	.163	.760	True	.797	.802
		IVW	13	1.095 (.985–1.217)	.054	.092		.852	
		Weighted median	13	1.137 (.994–1.301)	.074	.062			
EMS_total	CD	MR Egger	14	1.249 (.842–1.853)	.201	.291	TRUE	.212	.622
		IVW	14	1.134 (1.006–1.278)	.061	.040		.255	
		Weighted median	14	1.099 (.943–1.281)	.081	.227			
EMS (ASRM I/II)	IBD	MR Egger	8	.828 (.381–1.800)	.650	.650	True	.743	.488
		IVW	8	1.108 (1.015–1.209)	.045	.023		.773	
		Weighted median	8	1.079 (.961–1.211)	.059	.200			
EMS (ASRM I/II)	UC	MR Egger	8	.557 (.209–1.488)	.501	.288	True	.616	.196
		IVW	8	1.149 (1.029–1.284)	.057	.014		.476	
		Weighted median	8	1.174 (1.006–1.369)	.079	.042			
EMS (ASRM I/II)	CD	MR Egger	8	1.375 (.480–3.938)	.537	.575	True	.922	.608
		IVW	8	1.030 (.914–1.160)	.061	.628		.943	
		Weighted median	8	.969 (.836–1.123)	.075	.678			
EMS (ASRM III/IV)	IBD	MR Egger	19	1.270 (1.019–1.584)	.112	.048	True	.023	.776
		IVW	19	1.098 (1.019–1.184)	.038	.014		.047	
		Weighted median	19	1.118 (1.026–1.218)	.044	.014			
EMS (ASRM III/IV)	UC	MR Egger	19	1.162 (.863–1.564)	.152	.337	True	.023	.776
		IVW	19	1.115 (1.012–1.227)	.049	.027		.032	
EMS (ASRM III/IV)	UC	Weighted median	19	1.144 (1.029–1.272)	.0542	.016			
EMS(ASRM III/IV)	CD	MR Egger	19	1.352 (1.024–1.785)	.142	.049	True	.179	.121
		IVW	19	1.086 (.987–1.195)	.049	.090		.108	
		Weighted median	19	1.063 (.945–1.196)	.060	.310			
EMS_ovary	IBD	MR Egger	17	1.190 (1.001–1.415)	.088	.068	True	.155	.321
		IVW	17	1.093 (1.028–1.163)	.031	.005		.146	
		Weighted median	17	1.103 (1.023–1.190)	.039	.011			
EMS_ovary	UC	MR Egger	9	1.100 (.801–1.512)	.162	.575	True	.729	.955
		IVW	9	1.090 (.994–1.196)	.047	.068		.816	
		Weighted median	9	1.101 (.977–1.240)	.061	.114			
EMS_ovary	CD	MR Egger	18	1.283 (1.008–1.635)	.123	.060	True	.092	.139
		IVW	18	1.072 (.981–1.173)	.046	.126		.051	
		Weighted median	18	1.073 (.969–1.189)	.052	.177			
EMS_pelvic peritoneum	IBD	MR Egger	9	1.160 (.731–1.843)	.236	.549	NA	.644	.733
		IVW	9	1.068 (.990–1.153)	.039	.087		.730	
		Weighted median	9	1.062 (.960–1.175)	.052	.245			
EMS_pelvic peritoneum	UC	MR Egger	9	1.060 (.558–2.014)	.327	.863	NA	.299	.915
		IVW	9	1.099 (.996–1.212)	.050	.060		.394	
		Weighted median	9	1.086 (.959–1.230)	.063	.191			
EMS_pelvic peritoneum	CD	MR Egger	9	1.311 (.701–2.454)	.320	.424	NA	.980	.432
		IVW	9	1.008 (.909–1.118)	.053	.875		.972	
		Weighted median	9	.973 (.854–1.109)	0.066	.684			

Exposure	Outcome	MR methods	Number of SNPs	OR (95% CI)	SE	MR *p*‐Value	MR‐Steiger	*p*‐Value heterogeneity	*p*‐Value pleiotropy
EMS_rectovaginal septum and vagina	IBD	MR Egger	8	.729 (.467–1.139)	.227	.214	NA	.970	.128
		IVW	8	1.085 (1.018–1.157)	.033	.012		.726	
		Weighted median	8	1.079 (.990–1.175)	.044	.083			
EMS_rectovaginal septum and vagina	UC	MR Egger	8	.565 (.322–.991)	.286	.093	NA	.946	.051
		IVW	8	1.127 (1.036–1.226)	.043	.005		.368	
		Weighted median	8	1.107 (.995–1.231)	.054	.061			
EMS_rectovaginal septum and vagina	CD	MR Egger	8	1.139 (.621–2.091)	.310	.688	NA	.900	.725
		IVW	8	1.018 (.932–1.111)	.045	.697		.939	
		Weighted median	8	.987 (.887–1.099)	.054	.816			
EMS_occurring infertility	IBD	MR Egger	6	1.256 (.765–2.062)	.253	.418	NA	.766	.544
		IVW	6	1.065 (.970–1.170)	.048	.186		.810	
		Weighted median	6	0.990 (.846–1.158)	.080	.900			
EMS_occurring infertility	UC	MR Egger	6	1.538 (.825–2.864)	.317	.247	NA	.745	.288
		IVW	6	1.049 (.933–1.181)	.060	.423		.631	
		Weighted median	6	.990 (.846–1.158)	.080	.900			
EMS_occurring infertility	CD	MR Egger	6	1.150 (.543–2.435)	.383	.734	NA	.306	.922
		IVW	6	1.105 (.972–1.257)	.066	.127		.436	
		Weighted median	6	1.078 (.917–1.266)	.082	.363			

Abbreviations: ASRM, American Society for Reproductive Medicine; CD, Crohn's disease; CI, confidence interval; EMS, endometriosis; IBD, inflammatory bowel disease; IVW, inverse‐variance weighted; MR, Mendelian randomization; OR, odds ratio; SNP, single nucleotide polymorphism; UC, ulcerative colitis.

**TABLE 1D:** Causal estimation for the effect of EMS (Sapkota Y) on IBD, UC as well as CD.

Exposure	Outcome	MR methods	Number of SNPs	OR (95% CI)	SE	MR *p*‐value	MR‐Steiger	*p*‐Value heterogeneity	*p*‐Value pleiotropy
EMS_total	IBD	MR Egger	9	1.286 (.661–2.501)	.339	.483	True	.425	.917
		IVW	9	1.240 (1.087–1.415)	.067	.001		.532	
		Weighted median	9	1.245 (1.046–1.483)	.089	.014			
EMS_total	UC	MR Egger	9	1.429 (.505–4.045)	.531	.523	True	.144	.917
		IVW	9	1.323 (1.090–1.607)	.099	.005		.207	
		Weighted median	9	1.421 (1.125–1.796)	.119	.003			
EMS_total	CD	MR Egger	11	1.192 (.362–3.930)	.609	.780	NA	.039	.863
		IVW	11	1.072 (.863–1.330)	.110	.531		.059	
		Weighted median	11	.997 (.797–1.247)	.114	.979			

Abbreviations: CD, Crohn's disease; CI, confidence interval; EMS, endometriosis; IBD, inflammatory bowel disease; IVW, inverse‐variance weighted; MR, Mendelian randomization; OR, odds ratio; SNP, single nucleotide polymorphism; UC, ulcerative colitis.

It is the first MR study that analyzes the largest GWAS data of EMS patients available, providing robust evidence for detecting the causal estimates of IBD and EMS. Our findings suggest increased IBD risks following EMS, corroborating published research. Notably, our research contributes significant new insights to the field by unveiling the distinct effects of different EMS subtypes and stages on the risk of IBD. Owing to the multi‐system effects of EMS, the diagnoses are often delayed or misdiagnosed. We utilized genetic variants to identify genetic EMS, providing an alternative solution to this challenge.

The potential pathophysiological overlap between EMS and IBD may be a driving factor for the occurrence of IBD in EMS patients. Izumi et al. documented peritoneal immune features in EMS, noting abnormalities in neutrophils, macrophages, natural killer cells, and T/B lymphocytes.^11^ These immune dysregulations, associated with autoimmune diseases, have also been observed in IBD. Additionally, EMS involves excess production of prostaglandins, metalloproteinases (MMP), chemokines, and tumour necrosis factor‐alpha (TNF‐α), similar to indicators seen in IBD. Elevated MMP levels were observed in inflamed intestinal mucosa.^12^ Anti‐TNF therapy, an effective treatment for IBD, is also being explored for EMS treatment. Dysbiosis is a major risk factor for IBD and also closely correlates with EMS.^13^ These shared biological characteristics may provide some insight into the potential link between EMS and IBD, albeit with uncertain underlying mechanisms.

Although further validation is needed, this study contributes to optimizing the clinical management of EMS. First‐line therapy for EMS involves combining non‐steroidal anti‐inflammatory drugs (NSAIDs) with contraceptives or progestins.^14^ However, NSAIDs are involved in the development of IBD through various mechanisms, increasing the risk of UC (multivariate HR = 1.87) and CD (multivariate HR = 1.59). The use of contraceptives is also associated with a higher incidence of IBD (CD: RR = 1.46–1.51; UC: RR = 1.28–1.53).^15^ Alternative treatment options should be considered for EMS patients exposed to additional risks of IBD whenever possible. Gastrointestinal involvement can manifest in 5%−15% of EMS patients, showing symptoms like constipation, diarrhea, dysmenorrhea and dyspareunia, a colonoscopy is recommended to distinguish between IBD or EMS‐related intestinal manifestations. Tailored treatment is essential for EMS patients with coexisting IBD. For those without gastrointestinal symptoms, regular surveillance colonoscopies are beneficial.

Our research has certain limitations. While the outlier assessment and MR‐Egger test did not indicate any influence of pleiotropy on the results, it is possible that in certain unknown scenarios, complete correction of horizontal pleiotropy may not be achieved. On the other hand, the sample size of exposure and outcome are relatively small, we could not further distinguish the effect of EMS on two IBD subgroups.

In conclusion, our study confirmed the increased risk of IBD following EMS, but not vice versa.

## AUTHOR CONTRIBUTIONS

*Study concept and design*: Shutian Zhang. *Data acquisition and analysis*: Shutian Zhang and Yan Dang. *Drafting of the manuscript*: Yan Dang.

## CONFLICT OF INTEREST STATEMENT

The authors declare no conflict of interest.

## FUNDING INFORMATION

This research received no specific grant from any funding agency in the public, commercial or not‐for‐profit sectors.

## ETHICAL STATEMENT

Not Applicable.

## Supporting information

Supporting Information

Supporting Information

Supporting Information

Supporting Information

Supporting Information

Supporting Information

Supporting Information

Supporting Information

Supporting Information

## Data Availability

All data involved in the current study are publicly available data from individual referenced papers.
