# Supplementary figures and images for: Causal relationship between endometriosis and inflammatory bowel disease: A Mendelian randomization analyses
Source: Clin Transl Med. 2024 Jan 18;14(1):e1496. doi: 10.1002/ctm2.1496 (PMC10797250; doi:10.1002/ctm2.1496)

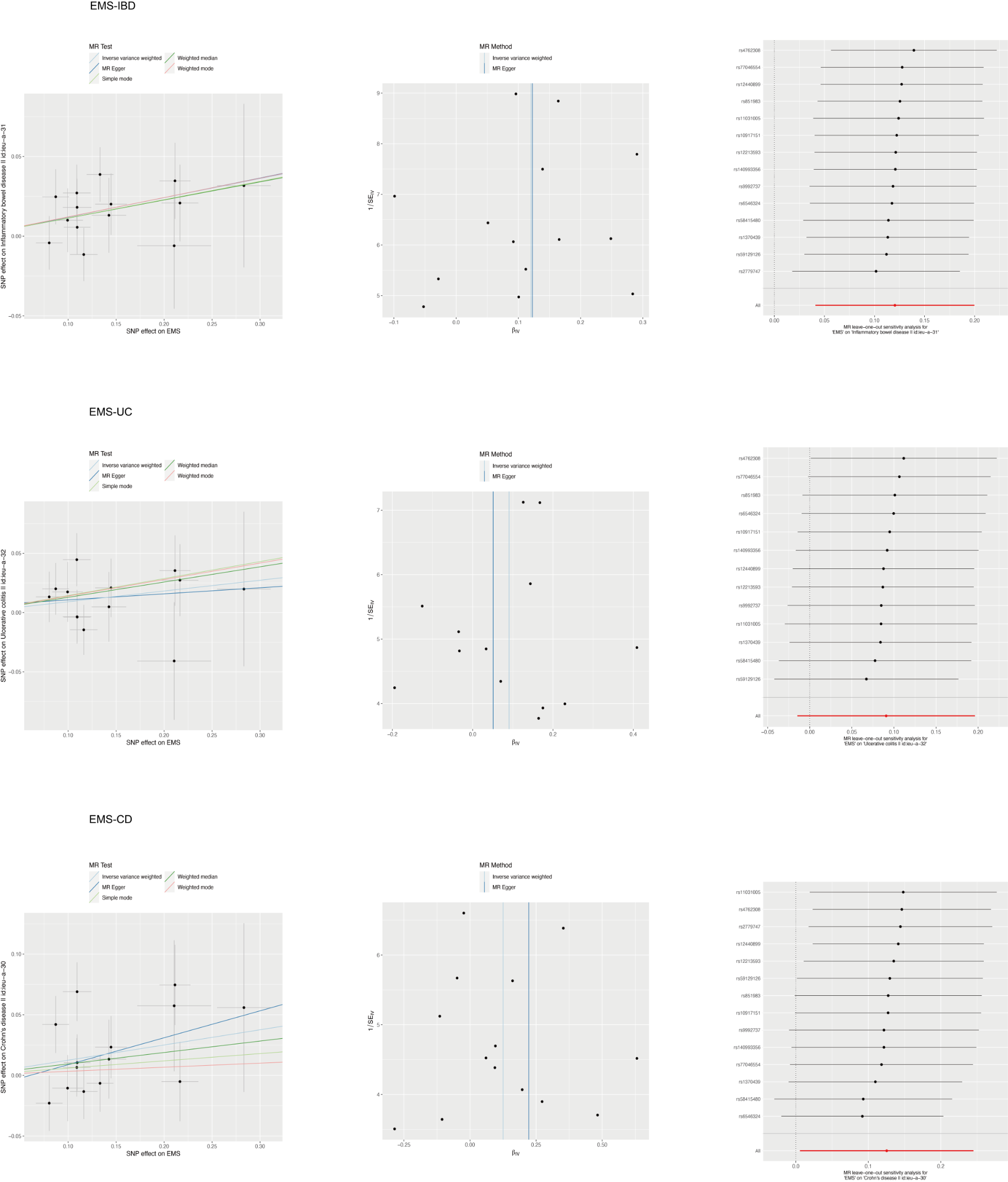

Supplement: Supplementary file 1 — Supporting Information [file CTM2-14-e1496-s007.tiff]

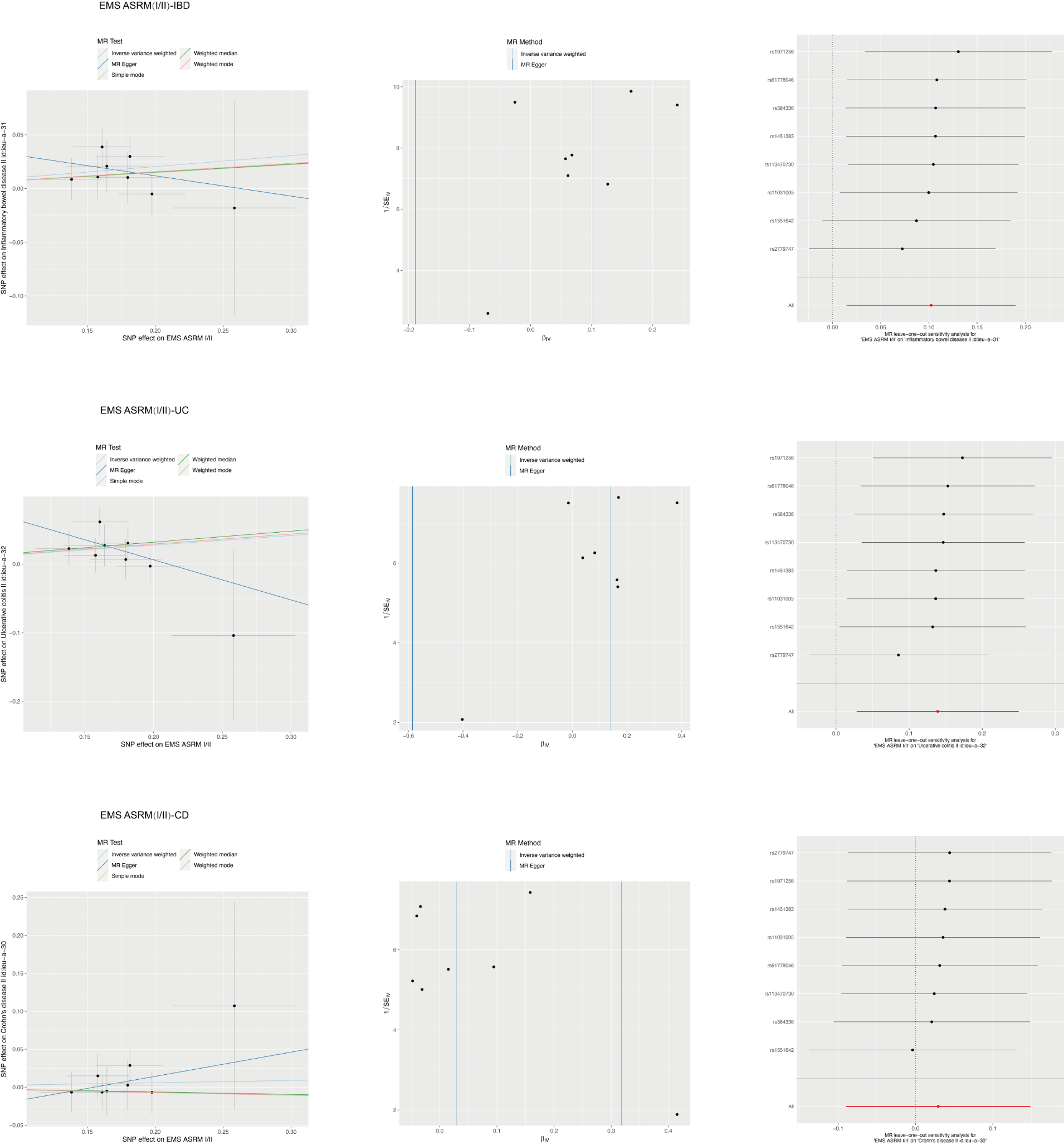

Supplement: Supplementary file 2 — Supporting Information [file CTM2-14-e1496-s005.tiff]

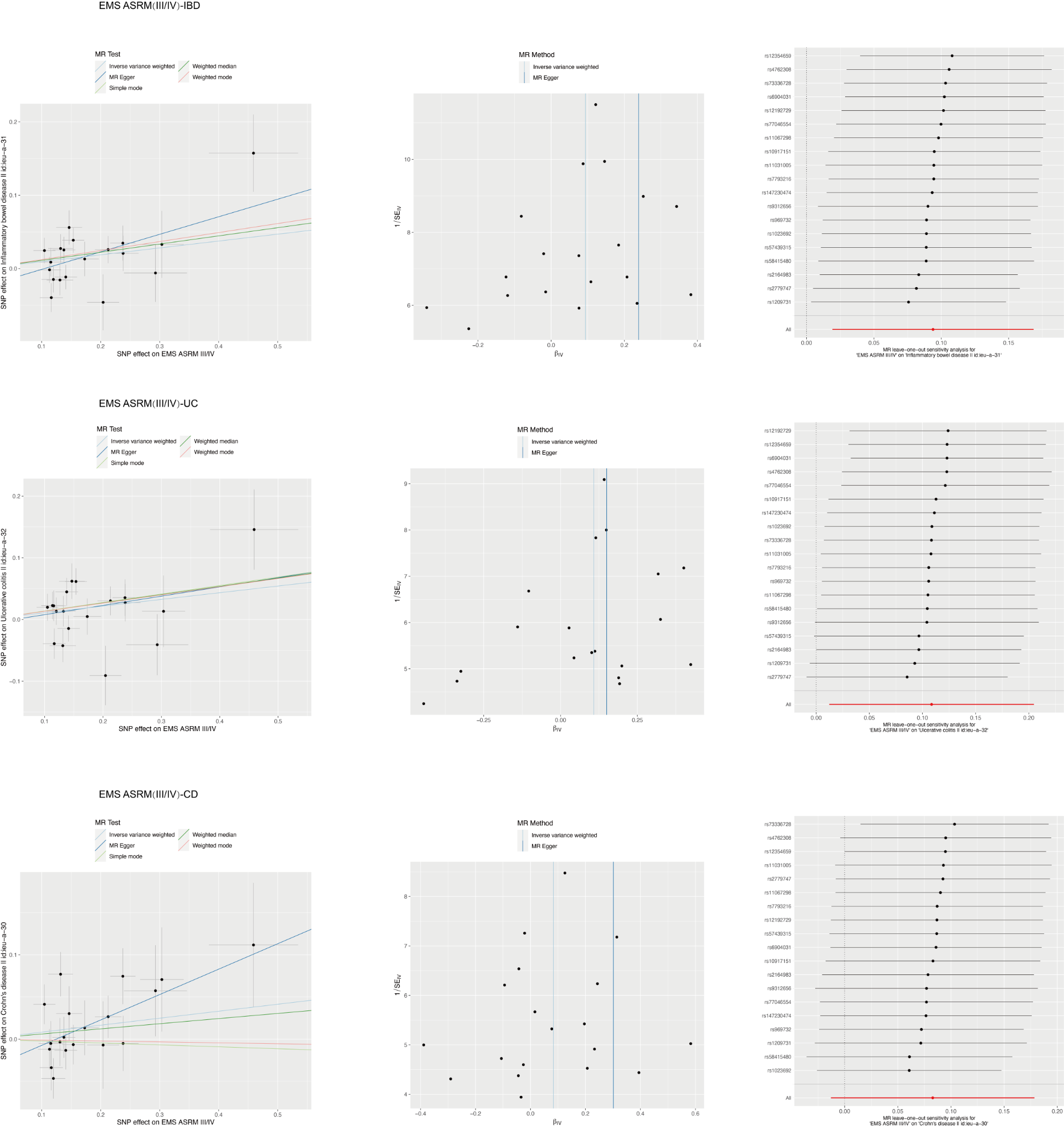

Supplement: Supplementary file 3 — Supporting Information [file CTM2-14-e1496-s006.tiff]

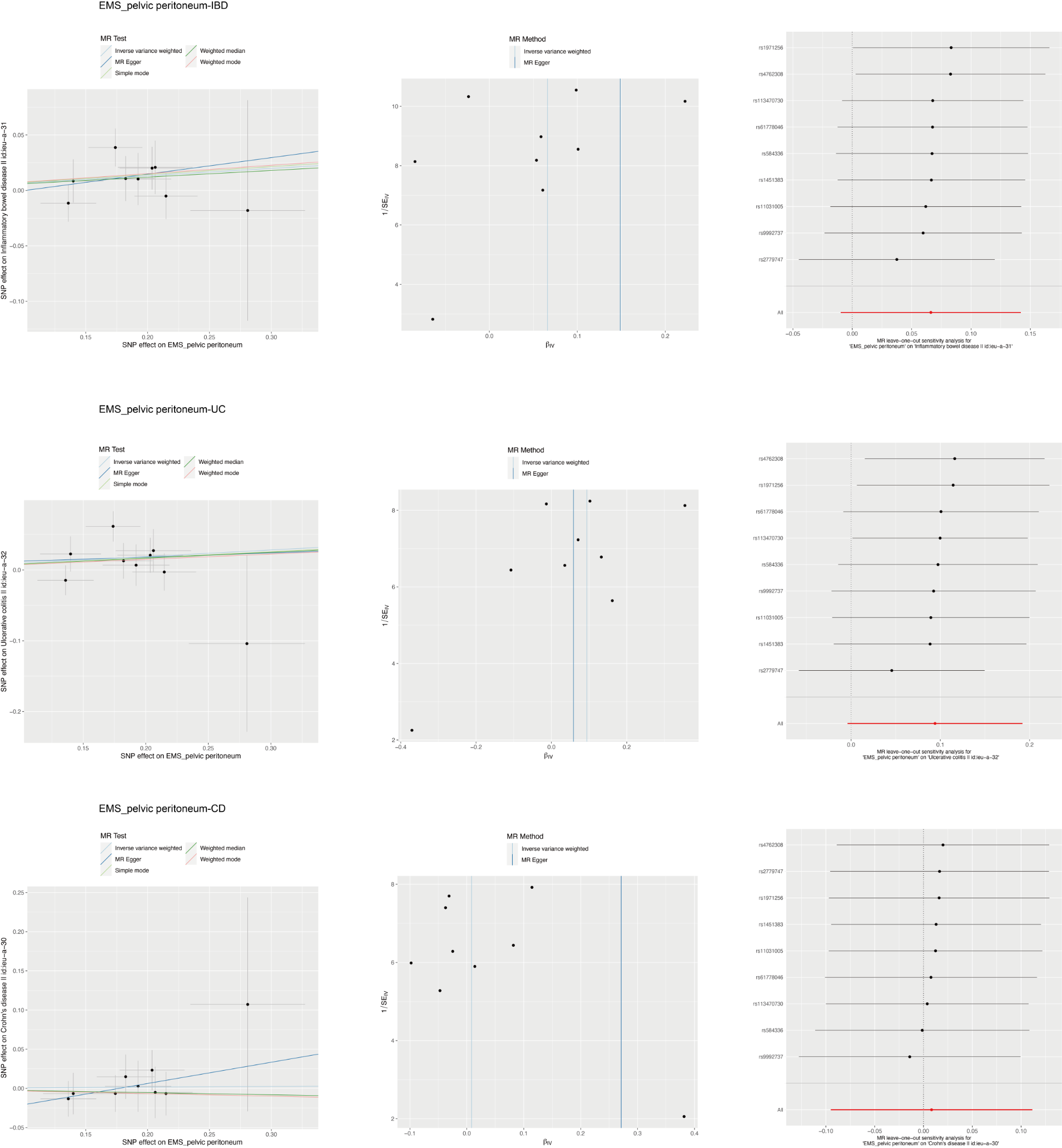

Supplement: Supplementary file 4 — Supporting Information [file CTM2-14-e1496-s002.tiff]

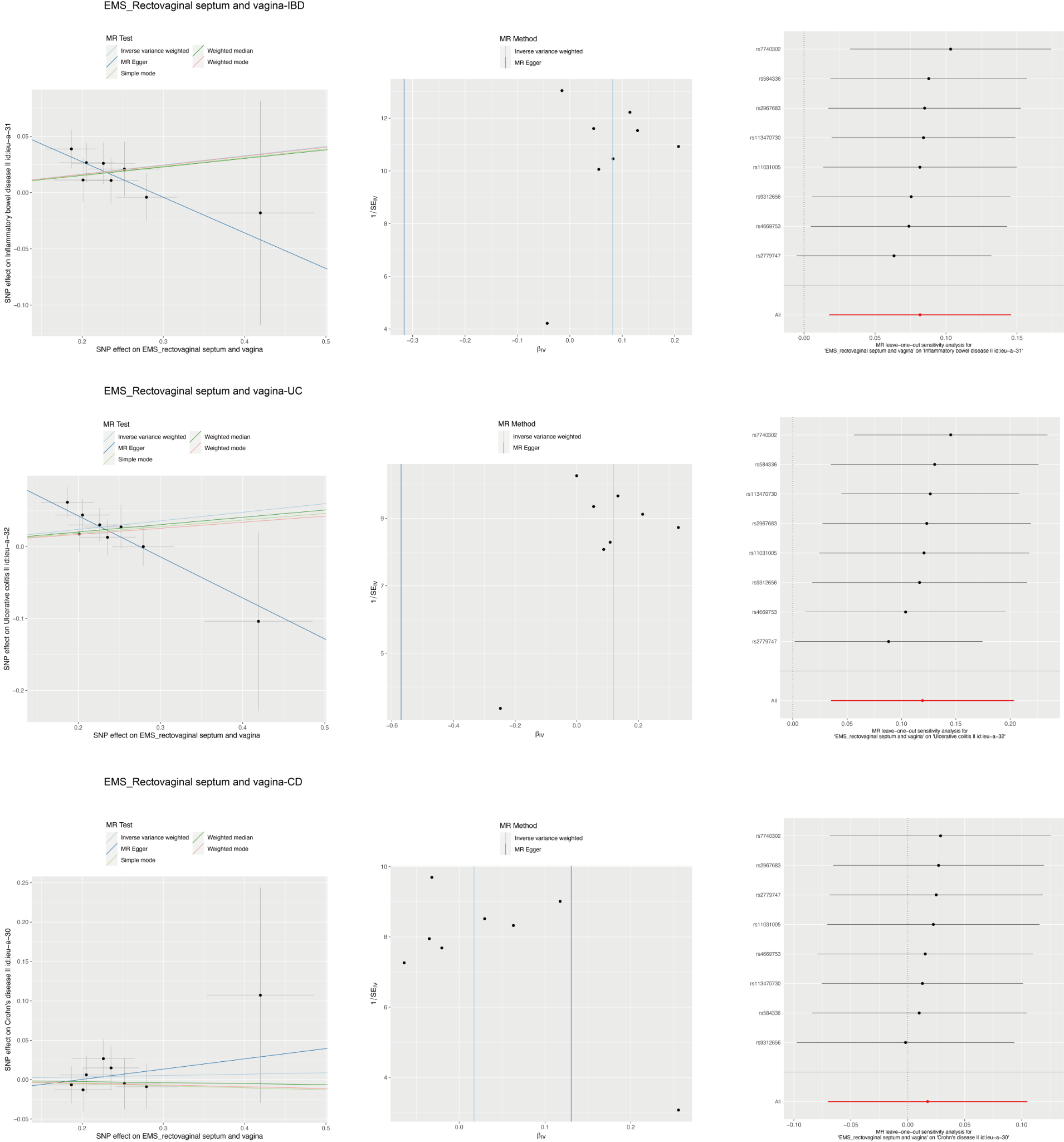

Supplement: Supplementary file 5 — Supporting Information [file CTM2-14-e1496-s003.tiff]

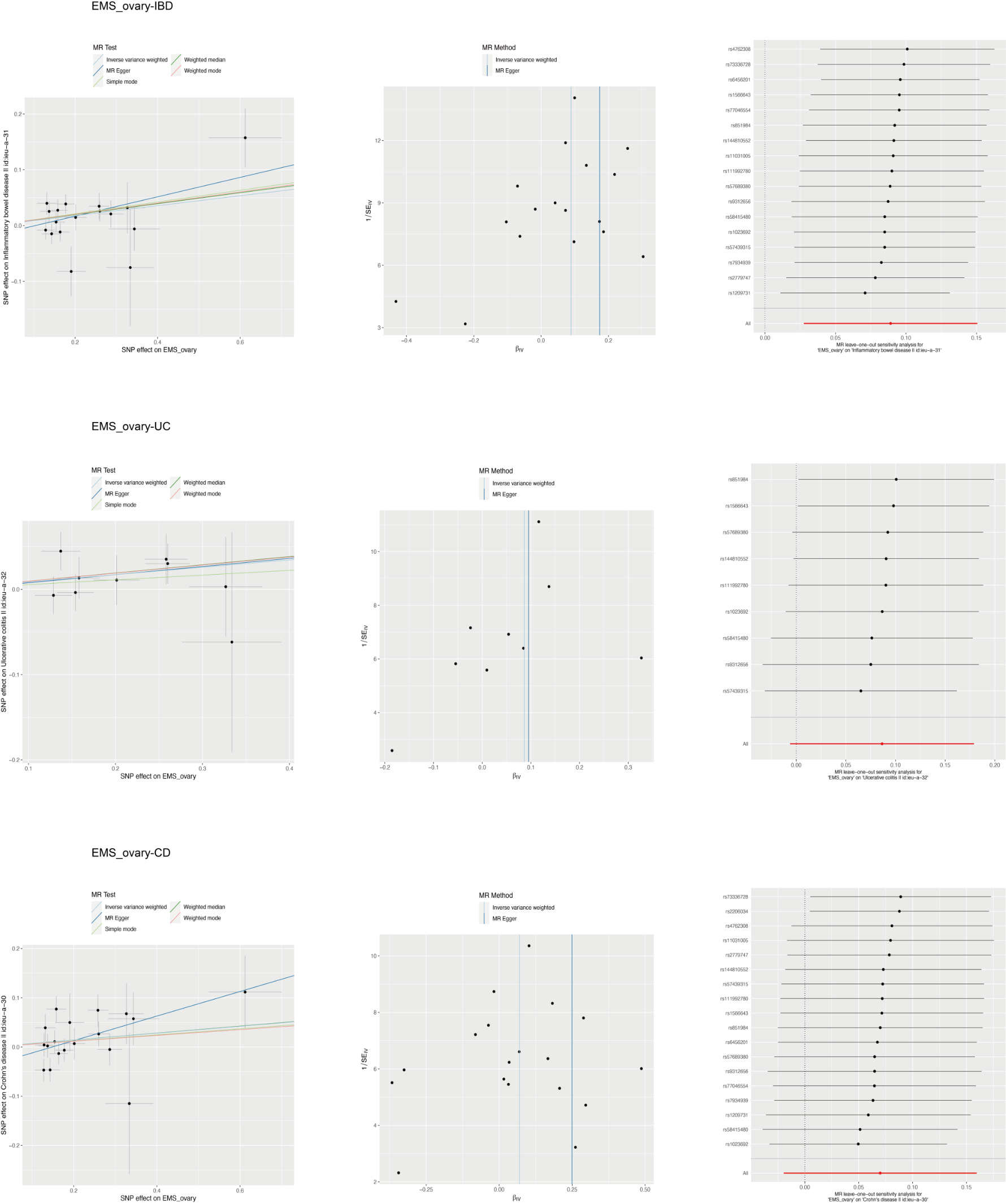

Supplement: Supplementary file 6 — Supporting Information [file CTM2-14-e1496-s004.tiff]

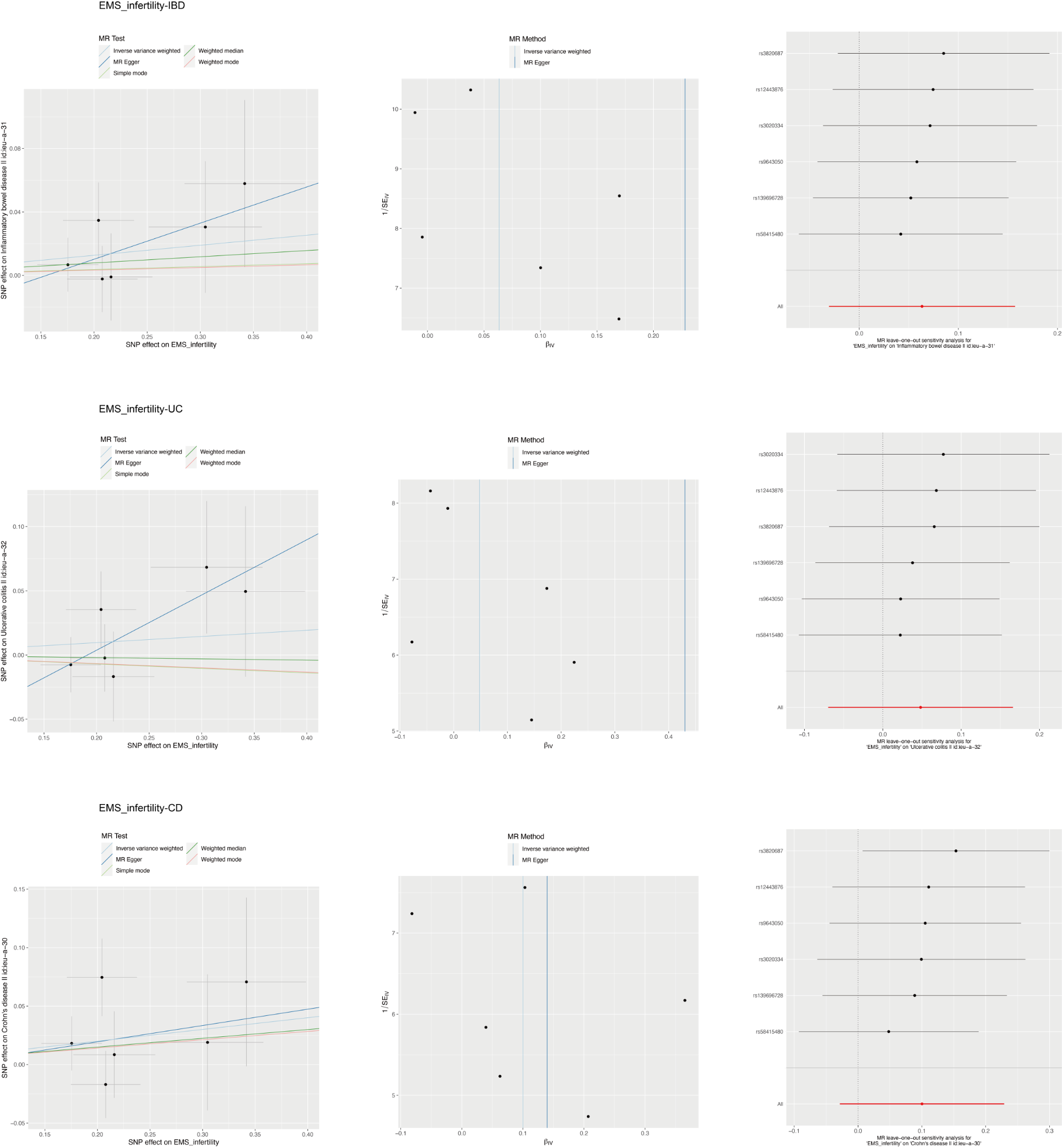

Supplement: Supplementary file 7 — Supporting Information [file CTM2-14-e1496-s009.tiff]
