# Supplementary material for: Causal relationship between endometriosis and inflammatory bowel disease: A Mendelian randomization analyses
Source: Clin Transl Med. 2024 Jan 18;14(1):e1496. doi: 10.1002/ctm2.1496 (PMC10797250; doi:10.1002/ctm2.1496)
Supplement: Supplementary file 9 — Supporting Information [file CTM2-14-e1496-s008.docx]

Supplementary Table 1: Summary of genetic instruments identified for MR Analyses.

Supplementary Table 2: Effect estimates of the associations of genetic instrumental variables for IBD and risk of EMS.

Supplementary Table 3: Statistical power of MR analysis.

**Table S1 Summary of Genetic Instruments identified for MR Analyses**

| **Exposure** | **Significant level** | **No. of SNPs** | **F statistics^†^** |
| --- | --- | --- | --- |
| **EMS** | 5e-08 | 14 | 52.913(24.521-123.038) |
| **EMS(ASRM III/IV)** | 5e-08 | 19 | 84.055(49.481-155.438) |
| **EMS_ovary** | 5e-08 | 18 | 68.874(34.999-141.464) |
| **EMS (Sapkota Y)** | 1e-05 | 11 | 62.051(39.820-105.417) |

Note: EMS, endometriosis; ASRM, American Society for Reproductive Medicine; **^†^**Median and range (minimum and maximum).

**Table S2 Effect estimates of the associations of genetic instrumental variables for IBD and risk of EMS**

| Exposure | Outcome | MR methods | Number of SNPs | OR (95% CI) | SE | MR  *p*-value | *p*-value  heterogeneity | *p*-value  pleiotropy |
| --- | --- | --- | --- | --- | --- | --- | --- | --- |
| IBD | EMS | MR Egger | 55 | 0.999 (0.919~1.085) | 0.042 | 0.974 | 0.250 | 0.776 |
|  |  | IVW | 55 | 1.010 (0.981~1.040) | 0.015 | 0.504 | 0.279 |  |
|  |  | Weighted median | 55 | 1.015 (0.973~1.060) | 0.022 | 0.488 |  |  |
| UC | EMS | MR Egger | 34 | 0.978 (0.884~1.081) | 0.051 | 0.661 | 0.774 | 0.422 |
|  |  | IVW | 34 | 1.017 (0.987-1.048) | 0.015 | 0.265 | 0.784 |  |
|  |  | Weighted median | 34 | 1.027 (0.984-1.071) | 0.022 | 0.225 |  |  |
| CD | EMS | MR Egger | 48 | 1.030 (0.967~1.097) | 0.032 | 0.364 | 0.122 | 0.314 |
|  |  | IVW | 48 | 1.000 (0.974~1.026) | 0.013 | 0.973 | 0.119 |  |
|  |  | Weighted median | 48 | 1.009 (0.972~1.047) | 0.019 | 0.635 |  |  |
| IBD | EMS(ASRM I/II) | MR Egger | 55 | 1.000 (0.882-1.134) | 0.064 | 1.000 | 0.289 | 0.708 |
|  |  | IVW | 55 | 1.023 (0.979-1.069) | 0.023 | 0.318 | 0.317 |  |
|  |  | Weighted median | 55 | 1.029 (0.964-1.098) | 0.033 | 0.391 |  |  |
| UC | EMS(ASRM I/II) | MR Egger | 34 | 1.037 (0.880-1.223) | 0.084 | 0.664 | 0.282 | 0.999 |
|  |  | IVW | 34 | 1.038 (0.989-1.089) | 0.025 | 0.134 | 0.325 |  |
|  |  | Weighted median | 34 | 1.040 (0.970-1.115) | 0.035 | 0.266 |  |  |
| CD | EMS(ASRM I/II) | MR Egger | 48 | 1.055 (0.967-1.151) | 0.044 | 0.235 | 0.483 | 0.136 |
|  |  | IVW | 48 | 0.992 (0.957-1.028) | 0.018 | 0.663 | 0.431 |  |
|  |  | Weighted median | 48 | 1.018 (0.964-1.076) | 0.028 | 0.519 |  |  |
| IBD | EMS(ASRM III/IV) | MR Egger | 55 | 1.031 (0.911-1.166) | 0.063 | 0.631 | 0.035 | 0.559 |
|  |  | IVW | 55 | 0.996 (0.953-1.040) | 0.022 | 0.849 | 0.039 |  |
|  |  | Weighted median | 55 | 0.973 (0.916-1.034) | 0.031 | 0.379 |  |  |
| UC | EMS(ASRM III/IV) | MR Egger | 34 | 1.010 (0.882-1.155) | 0.069 | 0.890 | 0.544 | 0.757 |
|  |  | IVW | 34 | 1.030 (0.990-1.073) | 0.021 | 0.143 | 0.589 |  |
|  |  | Weighted median | 34 | 1.038 (0.979-1.100) | 0.030 | 0.210 |  |  |
| CD | EMS(ASRM III/IV) | MR Egger | 48 | 1.030 (0.940-1.128) | 0.046 | 0.530 | 0.027 | 0.377 |
|  |  | IVW | 48 | 0.992 (0.956-1.029) | 0.019 | 0.655 | 0.027 |  |
|  |  | Weighted median | 48 | 0.981 (0.930-1.034) | 0.027 | 0.470 |  |  |
| IBD | EMS_ovary | MR Egger | 55 | 1.022 (0.894-1.168) | 0.068 | 0.750 | 0.122 | 0.489 |
|  |  | IVW | 55 | 0.978 (0.933-1.025) | 0.024 | 0.351 | 0.131 |  |
|  |  | Weighted median | 55 | 0.982 (0.917-1052) | 0.035 | 0.610 |  |  |
| UC | EMS_ovary | MR Egger | 34 | 0.991 (0.838-1.171) | 0.085 | 0.913 | 0.218 | 0.554 |
|  |  | IVW | 34 | 1.040 (0.990-1.093) | 0.025 | 0.121 | 0.240 |  |
|  |  | Weighted median | 34 | 1.022 (0.951-1.097) | 0.036 | 0.554 |  |  |
| CD | EMS_ovary | MR Egger | 48 | 1.032 (0.945-1.128) | 0.045 | 0.487 | 0.418 | 0.115 |
|  |  | IVW | 48 | 0.966 (0.931-1.002) | 0.019 | 0.065 | 0.356 |  |
|  |  | Weighted median | 48 | 0.986 (0.932-1.043) | 0.029 | 0.625 |  |  |

Note: MR, Mendelian randomization; IVW, inverse-variance weighted; OR, odds ratio; CI, confidence interval; SNP, single nucleotide polymorphism; IBD, inflammatory bowel disease; CD, Crohn’s disease; UC, ulcerative colitis; ASRM, American Society for Reproductive Medicine; EMS, endometriosis.

**Table S3 Statistical power of MR analysis**

| Exposure | Outcome | MR methods | Power (%) |
| --- | --- | --- | --- |
| EMS_total | IBD | MR Egger | 98 |
|  |  | IVW | 98 |
|  |  | Weighted median | 89 |
| EMS_total | UC | MR Egger | 35 |
|  |  | IVW | 82 |
|  |  | Weighted median | 99 |
| EMS_total | CD | MR Egger | 100 |
|  |  | IVW | 99 |
|  |  | Weighted median | 88 |
| EMS(ASRM III/IV) | IBD | MR Egger | 100 |
|  |  | IVW | 100 |
|  |  | Weighted median | 100 |
| EMS(ASRM III/IV) | UC | MR Egger | 100 |
|  |  | IVW | 100 |
|  |  | Weighted median | 100 |
| EMS(ASRM III/IV) | CD | MR Egger | 100 |
|  |  | IVW | 100 |
|  |  | Weighted median | 98 |
| EMS_ovary | IBD | MR Egger | 100 |
|  |  | IVW | 100 |
|  |  | Weighted median | 100 |
| EMS_ovary | UC | MR Egger | 99 |
|  |  | IVW | 98 |
|  |  | Weighted median | 99 |
| EMS_ovary | CD | MR Egger | 100 |
|  |  | IVW | 99 |
|  |  | Weighted median | 99 |
| EMS (Sapkota Y) | IBD | MR Egger | 100 |
|  |  | IVW | 100 |
|  |  | Weighted median | 100 |
| EMS (Sapkota Y) | UC | MR Egger | 100 |
|  |  | IVW | 100 |
|  |  | Weighted median | 100 |
| EMS (Sapkota Y) | CD | MR Egger | 99 |
|  |  | IVW | 50 |
|  |  | Weighted median | 39 |

Note: MR, Mendelian randomization; IVW, inverse-variance weighted; IBD, inflammatory bowel disease; CD, Crohn’s disease; UC, ulcerative colitis; ASRM, American Society for Reproductive Medicine; EMS, endometriosis.
